# Myelodysplastic syndromes: advantages of a combined cytogenetic and molecular diagnostic workup

**DOI:** 10.18632/oncotarget.16578

**Published:** 2017-03-25

**Authors:** Elena Ciabatti, Angelo Valetto, Veronica Bertini, Maria Immacolata Ferreri, Alice Guazzelli, Susanna Grassi, Francesca Guerrini, Iacopo Petrini, Maria Rita Metelli, Maria Adelaide Caligo, Simona Rossi, Sara Galimberti

**Affiliations:** ^1^ Department of Clinical and Experimental Medicine, Section of Hematology, University of Pisa, Pisa, Italy; ^2^ Laboratory of Medical Genetics, Azienda Ospedaliero-Universitaria Pisana, S. Chiara Hospital, Pisa, Italy; ^3^ GenOMec, University of Siena, Siena, Italy; ^4^ Department of Translational Research and New Technologies in Medicine, University of Pisa, Pisa, Italy

**Keywords:** myelodysplastic syndromes, aCGH, cytogenetics, TP53, SF3B1

## Abstract

In this study we present a new diagnostic workup for the myelodysplastic syndromes (MDS) including FISH, aCGH, and somatic mutation assays in addition to the conventional cytogenetics (CC). We analyzed 61 patients by CC, FISH for chromosome 5, 7, 8 and PDGFR rearrangements, aCGH, and PCR for ASXL1, EZH2, TP53, TET2, RUNX1, DNMT3A, SF3B1 somatic mutations. Moreover, we quantified WT1 and RPS14 gene expression levels, in order to find their possible adjunctive value and their possible clinical impact. CC analysis showed 32% of patients with at least one aberration. FISH analysis detected chromosomal aberrations in 24% of patients and recovered 5 cases (13.5%) at normal karyotype (two 5q- syndromes, one del(7) case, two cases with PDGFR rearrangement). The aGCH detected 10 “new” unbalanced cases in respect of the CC, including one with alteration of the ETV6 gene. After mutational analysis, 33 patients (54%) presented at least one mutation and represented the only marker of clonality in 36% of all patients. The statistical analysis confirmed the prognostic role of CC either on overall or on progression-free-survival. In addition, deletions detected by aCGH and WT1 over-expression negatively conditioned survival. In conclusion, our work showed that 1) the addition of FISH (at least for chr. 5 and 7) can improve the definition of the risk score; 2) mutational analysis, especially for the TP53 and SF3B1, could better define the type of MDS and represent a “clinical warning”; 3) the aCGH use could be probably applied to selected cases (with suboptimal response or failure).

## INTRODUCTION

Myelodysplastic syndromes (MDS) are a heterogeneous group of hematological disorders which involve hypercellular and dysplastic bone marrow (BM) and different grades of cytopenias in the peripheral blood (PB) [1]. The majority of patients affected by MDS are diagnosed during routine blood tests; symptoms of MDS are often secondary to the peripheral cytopenias caused by the bone marrow failure [2]. At the basis of diagnosis, is still today the cyto-morphological analysis of the PB as well as of BM and the bone marrow biopsy performance. The World Health Organization (WHO) recently revisited the MDS classification [3, 4], defining different entities according to the number of involved lineages (single or multiple), the presence of ring sideroblasts ( > 15% or < 5% in presence of the *SF3B1* mutations), and the blasts’ percentage. Moreover, also this classification does not still offer prognostic information, and thus different prognostic system scores help physicians to make correct therapeutic decisions. The international prognostic scoring system (IPSS) is still the most used prognostic tool, but a dynamic system score (WPSS) and the revised IPSS (R-IPSS) are also now available [2-5-6].

Conventional karyotyping continues to have a fundamental role in the classification and prognostication of MDS. Indeed, more than half of MDS patients present with one or more chromosomal aberrations; nevertheless, non-informative karyotypes still concern up to 20% of cases [7]. The integration of fluorescent hybridization *in situ* (FISH) allowed a more accurate definition of the chromosomal abnormalities offering the advantage of analyzing cells in interphase, but its role in the diagnostics of MDS it has not yet been well defined. First comparisons of cytogenetics and FISH indicated a role for FISH in facilitating MDS diagnosis and prognostication [8, 9]; nevertheless, other studies demonstrated an overlapping between conventional cytogenetics (CC) and FISH results, reserving the FISH analysis to those cases where karyotype failed [10].

In this complex scenario, the contribution of array CGH (Comparative Genomic Hybridization, aCGH) can overcome some of the limitations of the cytogenetic techniques: this method does not rely on cell division, has superb resolution for unbalanced lesions, and allows defining the copy number variation. Recent genome-wide microarray-based studies demonstrated a relevant frequency of submicroscopic copy number alterations in MDS, demonstrating that aCGH would be a potential method to detect cryptic relevant genomic markers [11, 12] revealing chromosomal aberrations in 80% of cases defined as “normal” by CC and FISH [13].

Another recent field of interest is the identification of somatic mutations, reported in a large variety of *de novo* MDS, including cases with normal cytogenetics [14]. Particular relevance had *EZH2, ASXL1, DNMT3A, IDH1/IDH2, SF3B1, TP53,* and *TET2*; *TET2* mutations were associated with better responses to 5-azacitidine [15]; *ASXL1*, *EZH2, TP53, and DNMT3A* mutations have been associated to worse outcome [16-18]. *IDH1* and *IDH2* mutations have been reported to impair the prognosis of patients with acute leukemia [19], but in MDS the prognosis seems to be not different [20]. In a large series of young patients affected by AML, Patel et al. identified at least one somatic alteration in 97.3% of them: *FLT3-ITD*, *MLL* partial tandem duplication, and *ASXL1* mutations were associated with reduced OS, whereas *CEBPA* and *IDH2* mutations were associated with improved OS. Interestingly, the favorable effect of *NPM1* mutations was restricted to patients with co-occurring *IDH1* or *IDH2* mutations [21].

On these basis, in the present study we set an integrated cytogenetic/molecular workup for the diagnosis of MDS in order to by-pass possible diagnostic failures and to increase the prognostic power of the today available diagnostic tools.

Thus, we screened 61 MDS patients for somatic mutations (*TET2, TP53, ASXL1, EZH2, RUNX1, DNMT3A, SF3B1*) by Real-Time PCR, in addition to the CC, FISH analysis, and aCGH. Finally, we performed quantitative RT-PCR assays to quantify the expression of two genes already known as prognostic in MDS: *WT1* and *RPS14.* The *WT1* (Wilms’ tumor) is a tumor suppressor gene [22] whose over-expression well correlates with blasts percentage [23, 24]. *RPS14* codifies for a ribosomal subunit; its haplo-insufficiency appeared to be responsible for the anemia in the 5q- syndrome [25], even if a reduced expression was also found in 71% of patients with an intermediate-1 IPSS risk without 5q- aberration [26].

Our results suggest the addition of some selected FISH and mutational assays in the diagnostic workup of MDS; probably, aCGH would be reserved to selected cases (where the CC fails or to resistant cases).

## RESULTS

### Patients

Clinical features of the 61 enrolled MDS patients are reported in the Table 1.

**Table 1:** Clinical features of enrolled patients.

Number of patients	61
**Median age**	74 years (range: 30-92)
**Sex**	M 71% F 29%
**Updated (2008)** **WHO classification** *Mufti GJ, et al.* *Haematologica. 2008.*	RA: 12% RARS: 15% RCMD: 33% RAEB 1-2: 21% 5q-: 3% MMCL: 12% n/a: 4%
**Updated (2016)** **WHO classification** *Arber DA., et al. Blood. 2016.*	MDS-SLD: 31% MDS-MLD: 8% MDS-RS-SLD: 13% MDS-RS-MLD: 2% MDS-EB 1-2: 28% MDS with isolated del(5q-): 3% MMCL: 12% n/a: 3%
**Hb value [g/dL]**	Median 11.2 (range 6.3-15.3)
**Neutrophils level [x10^9^/L]**	Median 2 (range 4.1-87)
**Platelets level [x10^9^/L]**	Median 100.5 (range 6-656)
**Erythopoietin level [mU/mL]**	Median 57 (range 23.4-137)
**Reticulocytes level [x10^9^/L]** **Reticulocytes %**	Median 56.279 (range 35.34-74.13) Median 1.4 (range 0.21-3.9)
**Ferritin level [ng/mL]**	Median 239.5 (range 60-2065)
**Blast (%)**	Median 5 (range 0-23)

According to the IPSS score, 67% of cases resulted at low/intermediate-1 and 26% at intermediate-2/high, while according to the R-IPSS 49% were at very-low/low, 33% at intermediate, and 18% at high/very-high risk.

### Conventional cytogenetics

CC analysis was performed in all patients; in 12% of them it was not possible to analyze 20 metaphases, so CC failed. CC analysis documented in 32% of patients at least one chromosomal aberration, such as trisomy of chromosome 8 (in 9% of cases), deletion of chromosome 5 (in 4%), of chromosome 7 (in 2%) or of the Y (in 2%). Complex karyotype was found in 7% of patients, and in single cases deletion of chromosome 13, duplication of chromosome 14, t(9;12) (q32;q12), and trisomy of chromosome 6 have been observed. Overall, these results significantly correlated with the WHO classification: 87% of the forms with excess of blasts presented an abnormal karyotype, followed by the unilineage cytopenia with ring sideroblasts (40%), and by the multi-lineage cytopenias (27%). All patients with unilineage cytopenia (previously classified as refractory anemia) had a normal karyotype.

### FISH

FISH for chromosomes 5, 7, 8 and PDGFR rearrangements was performed in all patients with normal karyotype and in the cases who failed CC, in order to identify eventual “new” chromosomal aberrations. In cases with abnormalities of chromosome 5, 7 or 8 at the CC, FISH was used for confirming results. FISH analysis failed in only 2 cases, and it was not be deliberately performed in the 4 cases with complex karyotype; thus, its applicability resulted of 96.5% *versus* 88% of the CC. FISH results were available for the 37 patients with normal karyotype; in 5 cases (13.5%) FISH allowed us identifying a new chromosomal abnormality: in 2 cases a 5p15.2-q31 deletion, in one a deletion of chromosome 7, and in other 2 patients a *PDGFRB* rearrangement. In these cases, in order to exclude a myeloid disorder with *PDGFR* rearrangement, a PCR for the *PDGFRB-ETV6* fusion gene was performed, but it resulted negative. In cases where the FISH analysis was adopted for confirming the results from the CC, a full concordance was observed. Overall, by adding the chromosomal abnormalities detected by FISH to those already shown by CC, the 51% of our patients presented at least one chromosomal aberration.

### aCGH

aCGH analysis was performed in 59 patients; in the 2 remaining cases it failed for insufficient available DNA. Overall, copy number changes were identified in 23 cases (39%): 14 showed deletions involving chromosomes 3, 5, 7, 8, 11, 12, 13, 16, 17, and 20, and one patient presented loss of chromosome Y. Three cases harbored gains of chromosome 8, and other 3 showed a duplication of chromosome 1, 3, and 14, respectively. The last two cases harbored a complex karyotype: one involved chromosomes 1, 4, 7, 17 and 21, and the other one showed a deletion of 16q23.2 added to a mosaic of two different mutation of chromosome 11. In 13 patients, aCGH confirmed the cytogenetic analyses (4 and 5 cases confirmed the CC and FISH results, respectively); in the 10 remaining cases (43.5%), already defined as “normal”, aCGH identified “new” copy number alterations (Table 2). Interestingly, the 12p13.2 breakpoint fell into the *ETV6* gene, which results partially deleted; this type of deletion has been detected in a broad spectrum of hematological malignancies, frequently with monosomy 7, such as in our case [27]. All comparative results are reported in Table 3.

**Table 2:** Chromosomal “new”abnormalities detected by CGH array.

n° pts	Diagnosis	aCGH data	Affected genes
Cases with submicroscopic gains (118 Kb)			
1	RCMD/MDS-SLD	arr 3q25.32(158422273-158540638)x3	RARRES1/MFSD1
1	RARS/MDS-RS-SLD	arr 1q25.1(173884057-174002363)x3	SERP/RC3H1
Cases with submicroscopic losses (range: 2.9 Kb-46 Mb)			
1	RCMD/MDS-SLD	arr 20p12.1(14875511-15199538)x1	MACROD2 (324Kb)
1	RCMD/MDS-SLD	arr 16q23.2(79627703-79630630)x1	MAF (2.9 Kb)
1	CMML	arr 3q21.2.q21.3(125672997-128686163)x1	GATA2 (3Mb)
1	RAEB 2/MDS-EB 2	arr 3p26.1(4134024-4505821)x1	SETMAR /SUMF1 (371kb)
1	CMML	arr 17p13.3(3466507-3490413)x1	CARKL (23 Kb)
1	RCMD/MDS-SLD	arr Yp11.32-q11.23(869217-28660577)x0	
1	RARS/MDS-RS-SLD	arr 11q14.1-q24.3(83033530-129361159)x1	ANO3/MUC15 (46Mb)
1	CMML	arr 12p13.2-p11.23(11897416-26698489)x1	ETV6 (14,68Mb)

**Table 3:** Conventional cytogenetics, FISH, aCGH and somatic mutations comparative results.

CC (7 failures)		FISH (2 failures) (4 CK not done)		CGH array (2 failures)		Somatic Mutations [ASXL1, EZH2, TP53, TET2, RUNX1, DNMT3A, SF3B1 gene]	
abn	normal	abn	normal	abn	normal	abn	normal
17 cases	37 cases	13 cases	42 cases	23 cases	36 cases	33 cases	28 cases
		8 previously identified cases		13 previously identified cases		11 previously identified cases	
		5 “new detected” cases		10 “new detected” cases		22 “new detected” cases	

### Somatic point mutation analysis

Somatic point mutations analysis for *TET2, EZH2, TP53, ASXL1, SF3B1, RUNX1, DNMT3A* was performed in all cases; 33 patients (54%) presented at least one mutation. The more frequent mutations resulted *TP53* in 18 cases (29.5%) and *SF3B1* in 12 patients (19.7%). *ASXL1* was mutated in 5 patients (8%), *TET2* in 2 cases (3.3%), *RUNX1* in only one case (1.6%), and no cases showed *DNMT3A* or *EZH2* mutations. In the cohort of patients with normal karyotype, *TP53* was mutated in 25% of them, *SF3B1* in 22%, ASXL1 in 11%, *TET2* and *RUNX1* in 2.7% of cases.

*TP53* mutation interested 29.4% of the multi-lineage cytopenias, 26.7% of the forms with excess of blasts, 25% of the 5q-, and 23.5% of the unilineage cytopenia and chronic myelo-monocytic leukemia. Ten of the 12 patients with ring sideroblasts (83.3%) presented the *SF3B1* mutation; in 5 cases (41.6%), *SF3B1* was concomitant to the *TP53* mutation. In the remaining cases, mutations were exclusive.

No correlation were observed between somatic mutations and risk scores, except for the *SF3B1* that was mutated in 30% of the cases in the low/very low *vs* 20% in the high/very high risk WPSS category (*p* = 0.048). No differences in respect of age or sex were found. In 3 of our patients, a different neoplasia was reported; in these cases, we cannot be sure that the mutation detected was really somatic and not the expression of a germ-line predisposition; unfortunately, no fibroblasts or other somatic sources were available for comparison.

### WT1 and RPS14 gene expression

The *WT1* results were expressed as *WT1* copies/*ABL* x 10^4^ copies and the normal ratio was considered from 3 to 180 copies. Patients with high WT1 expression were 28% of the all patients, with a value ranging from 242 to 3395.

*RPS14* quantitative results were normalized and expressed as *RPS14*/*18S* copies ratio. The statistical analysis was performed stratifying samples in “low” and “high” respect to the median values measured in the healthy donors (median = 1,073). The majority of cases showed low levels of *RPS14* gene expression, while the high-level cases were 11% of the all patients (range 1.07-1.47).

*WT1* or *RPS14* expression did not differ according to the karyotype (normal *vs* abnormal), IPSS, WPSS or R-IPSS scores. Moreover, there was not correlation between genes’ expression values and somatic mutation; on the contrary, higher WT1 levels were found in patients with chromosomal deletions shown by aCGH in respect of no deleted cases (58.3% *vs* 18.7%; *p* = 0.023).

### Clinical outcome

Overall, 52 patients were enrolled concomitantly to the diagnosis of MDS, whereas in other 9 the diagnosis of MDS has been performed previously, with a median interval of 32 months (range: 11-56 months) to the accrual. In these cases, cytogenetic and molecular analyses were performed in occasion of a routine bone marrow assessment.

With a median follow-up of 21 months (range: 8-41), 67% of patients were still alive at 30 months; when patients were stratified on the basis of treatment, median OS was 33 months for patients receiving erythropoietin (Epo), granulocyte stimulating factor (G-CSF) or supportive care, compared to 37 months for those treated with azacitidine, and 21 months for patients affected by chronic myelomonocytic leukemia (CMML) or 5q- syndrome that received hydroxyurea or lenalidomide. These observed differences were not statistically significant.

Progression-free-survival (PFS) was calculated from the eighth week of treatment with Epo or from the forth month of therapy with azacitidine in cases with stable disease or from the date of best response (for responsive cases) until the day of progression into acute leukemia. If deaths occurred for causes not disease-related, patients were considered as “progression free”. For the entire series, median PFS was not reached during the observation interval; at 30 months, 78% of patients still did not progress. When patients were stratified on the basis of treatment, 30-months PFS was 85% for patients receiving erythropoietin or G-CSF or supportive care, *versus* 87% of those treated with azacitidine, *versus* 55% of patients receiving hydroxyurea or lenalidomide. As for OS, also these differences were not significant.

On the contrary, median OS was longer for patients achieving a good response [hematological improvement (HI), partial (PR) or complete response (CR)] after treatment in comparison to those with stable (SD) or progressive disease (PD) (44 *versus* 28 months; *p* = 0.015). Also the percentage of blasts measured in the bone marrow smears at the enrollment was predictive of OS: patients carrying ≥10% blasts showed a shorter median OS (18 months *versus* 41; *p* = 0.009).

Then, OS was analyzed according to the IPSS, WPSS, and R-IPSS risk scores: IPSS and WPSS were able to significantly categorize the different risk groups also in our series (*p* = 0.03; *p* = 0.01); about R-IPSS, the statistical power was not reached (*p* = 0.12), even if a trend for a shorter survival was observed for high and very-high risk patients.

**Figure 1:**
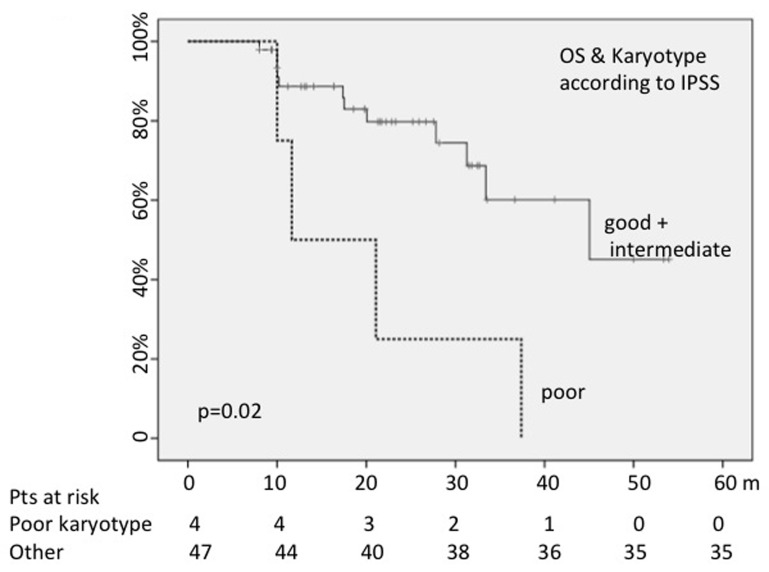
Poor karyotype negatively conditioned OS

When we analyzed the OS probability on the basis of karyotype risk (23% or 21% with poor karyotype according to the IPSS or the R-IPSS scores), cases with higher cytogenetic risk showed a shorter OS (median = 11 months *versus* 33 with IPSS score: *p* = 0.02; 10 months *versus* 34 with R-IPSS score: *p* = 0.02) (Figure 1).

The presence of chromosomal aberration detected by CC or by FISH did not impact on OS.

**Figure 2:**
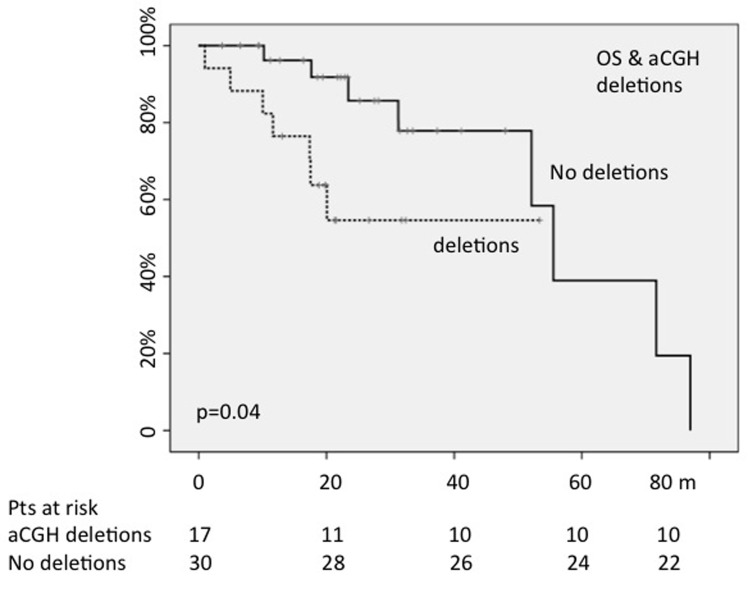
The presence of chromosomal deletions, detected by array CGH, negatively conditioned OS (OS:30-months; p=0.04)

The presence/absence of whatever abnormality found by aCGH did not change OS; on the contrary, 30-months OS was 57% for patients showing a deletion shown by aCGH *versus* 83% of not deleted patients (*p* = 0.04) (Figure 2).

When we analyzed the impact of deletions detected by aCGH according to the karyotype (normal *vs* abnormal), we found that the 30-months OS was 85% for cases with normal karyotype/no deletions by aCGH *vs* 54% for those with normal karyotype plus deletions; nevertheless, this difference of 31% did not reach the statistical significance (*p* = 0.30). On the contrary, in the subgroup with abnormal karyotype, the 18-months OS (in this cohort the median follow-up was shorter) was 100% for cases without deletions by aCGH *vs* 50% for those with deletions; this difference was significant (*p* = 0.025), thus suggesting that a particular attention (and then a more strict follow-up) has to be done in cases with deletions detected by aCGH, especially in cases with abnormal karyotype.

When Real-Time PCR tests were performed in order to find gene mutations, the OS analysis between mutated and wild-type patients did not detect significant differences, neither in the subgroup with normal, nor in that with abnormal karyotype. Only in the cohort with normal karyotype, the *SF3B1* mutations positively conditioned the 30-months PFS, even if without statistical significance (100% for mutated cases *vs* 79% for the wild-type ones; *p* = 0.22).

**Figure 3:**
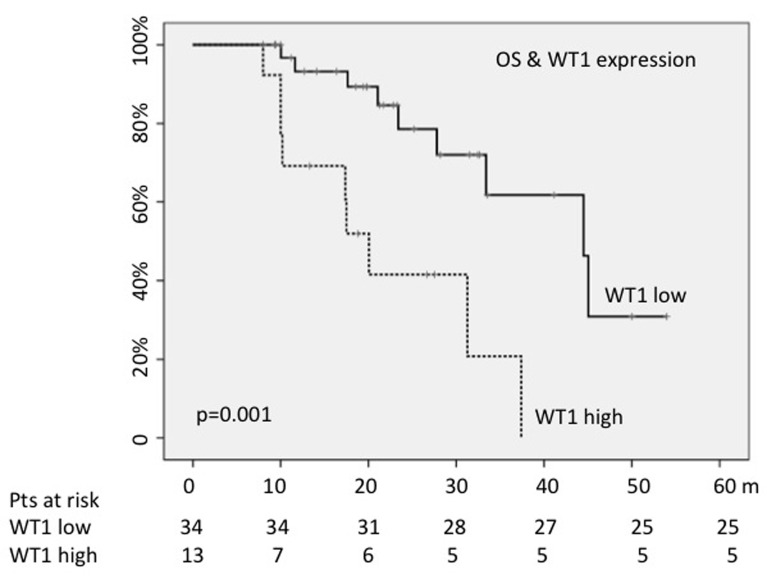
WT1 high levels predicted shorter OS

Finally, OS was evaluated according to *WT1* and *RPS14* values; at the enrollment, 70% of all patients showed *RPS14* values lower than those measured in healthy donors; on the other hand, 28% carried higher *WT1* levels. *RPS14* expression levels did not condition median OS; on the contrary, OS resulted shorter for cases showing high *WT1* values (18 months *versus* 41; *p* = 0.001) (Figure 3). The high *WT1* expression levels maintained a statistical significance also in multivariate analysis, including blasts number, quality of response, risk scores and chromosomal deletions (HR = 3.78; 95%CI = 1.9-1037; *p* = 0.018).

In the overall series, PFS was not significantly affected by sex, age > 65 years, or type of treatment, even if it resulted 16 months shorter for older patients in respect of the younger ones. On the contrary, 30 months-PFS was higher for patients achieving clinical response after treatment in comparison to those with stable or progressive disease (100% *versus* 67%; *p* = 0.002). Differently from that already observed for OS, ≥10% blasts did not significantly impact on PFS, even a trend for longer progression-free time was observed for cases with < 10% blasts (*p* = 0.06). The PFS was analyzed according to the IPSS, WPSS, and R-IPSS risk scores categories and only the WPSS score was able to significantly categorize the different risk groups (*p* = 0.01). When PFS was analyzed according to cytogenetic features, the presence/absence of chromosomal aberrations did not result significant.

On the contrary, as expected, cases at higher cytogenetic risk, either classified according to the IPSS or the R-IPSS scores, showed a lower 30-months PFS rate (37% *versus* 83%; *p* = 0.01). Even when FISH results (molecular abnormalities present/absent) were computed, no differences in PFS were observed.

Then, we analyzed the prognostic role of chromosomal abnormalities detected by aCGH; when the whole population was considered, no differences in PFS were observed. Then, as for the OS, we analyzed the impact of deletions detected by aCGH according to the karyotype (normal *vs* abnormal); we found that the 30-months PFS was 86% for cases with normal karyotype/no deletions by aCGH *vs* 71% for those with normal karyotype plus deletions; nevertheless, this difference of 15% did not reach the statistical significance (*p* = 0.64). On the contrary, in the subgroup wit abnormal karyotype, the 18-months PFS was 100% for cases without deletions by aCGH *vs* 57% for those with deletions; this difference was at the limit of the statistical significance (*p* = 0.060).

When PFS was measured according to mutations detected by PCR (*TET2, EZH2, TP53, ASXL1, SF3B1, DNMT3A, RUNX1*), no significant differences were detected between the subgroup with normal and that with abnormal karyotype.

**Figure 4:**
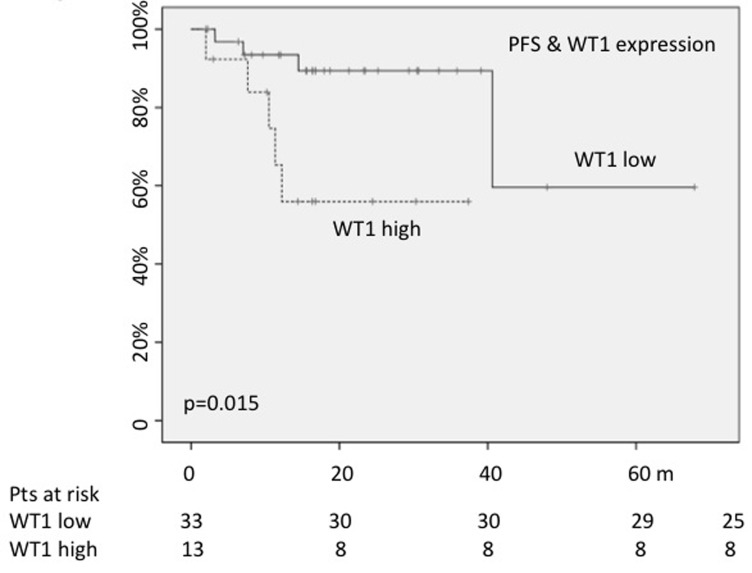
High WT1 expression played a negative impact on PFS

Finally, PFS was estimated according to the *WT1* and *RPS14* values. As described for the analysis of the OS, *RPS14* expression was not significant, whereas 30-months PFS rate resulted significantly lower for cases carrying *WT1* higher values (56% *versus* 89%; *p* = 0.01) (Figure 4).

When multivariate analysis was performed including quality or response, WPSS risk score, poor karyotype, and *WT1* levels, none of the considered variables retained their prognostic significance.

## DISCUSSION

As above reported, more than half of MDS cases present with abnormal karyotype; nevertheless, non-informative karyotypes represent up to 20% of cases. In this way, the integration of molecular techniques (FISH, aCGH, PCR) are promising tools for a more accurate definition of the chromosomal abnormalities and then for a more correct risk stratification. Thus, in our study 61 MDS patients were analyzed of diagnosis by conventional cytogenetics, FISH, aCGH and mutational analysis by Real-Time PCR.

As first finding, in our study a lower rate of CC failure was observed (only 12%); this low percentage of failures in comparison to rates reported in literature would depend from a more correct management of samples. Indeed, we used for cytogenetic analyses the bone marrow samples aspirated immediately after doing the bone marrow smears destined to the morphological assessment. Overall, 55% of our patients presented with chromosomal abnormalities (detected by CC and/or FISH). The relevance of conventional cytogenetic analysis in the clinical practice is evident also in our series: the high cytogenetic risk score was associated with a shorter OS (median: 11 months *vs* 33 according IPSS: *p* = 0.02; 10 months *versus* 34 according R-IPSS: *p* = 0.02) and PFS (at 30 months, 37% *versus* 83% free of progression into acute leukemia; *p* = 0.01).

After the FISH analysis, five “new” chromosomal abnormalities were observed, thus recognizing to the FISH an additive value in respect of the CC analysis. In particular, FISH allowed us correctly classifying two patients with normal karyotype as affected by the 5q- syndrome; in another case, the identification of deletion of chromosome 7 allowed us inserting our patient into the poor cytogenetic risk group, so offering to them the correct treatment (lenalidomide and azacitidine, respectively). Finally, two patients presented the *PDGRFbeta* rearrangement, and thus they received imatinib, effective in MDS cases with PDGFR rearrangement in addition to the chronic myeloid leukemia [29]. Both patients maintained for several months a partial response and then underwent successfully to the allogeneic transplantation. Recently, the advantages of FISH for abnormalities of chromosome 8, 7, 5, 20, and Y in respect of CC has been reported in a very huge series by a Chinese group: conventional banding was successful in 94.0% of patients, whereas FISH resulted informative in 100% of cases. Interestingly, FISH identified abnormalities in 50% of cases where the CC failed, and in 24% of the patients exhibiting normal karyotype. Authors concluded that FISH had to be applied to cases where the CC failed or with a normal karyotype [30]. The advantage given by the FISH addition in the Chinese study was higher than that found in our cohort (8.7%), but it would be depend either from the very different number of patients (more than 2000 *vs* 57) or from the fact that Chinese authors adopted also FISH for the chromosomal Y that we did not used. It is to be considered that this deletion can be detected in 5% of the healthy old male subjects [31], in cases affected by autoimmune disorders [32], neurodegenerative diseases [33], or neoplasia [34].

In conclusion, our results sustain what recently suggested by the European Leukemia Network, to add FISH in case of CC failure [4]; indeed, in our study we demonstrated that FISH analyses could represent an adjunctive value during the diagnostic phase of MDS, allowing us to correctly treat 3 cases.

About aCGH, we found chromosomal aberrations in the 17% of patients with normal karyotype, including two types of duplications and seven different losses of chromosomal regions. In particular, one case showed a partial deletion of 12p13.2 region in correspondence of the *ETV6* gene, associated with a partial monosomy of chromosome 7. In this patient, aCGH confirmed the monosomy of chromosome 7 and detected a chromosome 12p interstitial deletion not visible at the CC. Interestingly, the 12p13.2 breakpoint fell into the *ETV6* gene, which resulted partially deleted; *ETV6* is considered the main candidate tumor suppressor gene within this region [35-36]. Our data about aCGH reflect those already published by other groups: in a recent study, aCGH detected copy number changes in 11% of MDS patients with normal CC; the authors described recurrent sub-microscopic deletions, which encompassed some genes involved in MDS prognosis, such as *TET2* and *ETV6* [37]. In our cohort, aCGH allowed us identifying two different cases with deletion of the long arm of the chromosome 3 (one of these involving the *GATA2* gene); to note that both patients had normal CC and FISH. Abnormalities involving chromosome 3q are not frequent in MDS, but they have been associated with poor cytogenetic risk and shorter OS [39]; interestingly, both our patients developed acute leukemia during the study. Moreover, another interesting finding is that the negative prognostic impact is reserved to the deletions only: while the presence/absence of whatever abnormality detected by aCGH did not impact on OS, 30-months OS was 57% for patients with deletions detected by aCGH *versus* 83% of those without abnormalities or aberrations different from deletions (*p* = 0.04). These results are perfectly in line with those published by Volkert et al. who reported that aCGH detected copy number changes in 11% of MDS patients with normal karyotype and that OS was significantly shorter in patients with deletions detected in comparison to cases without abnormalities or other changes detected by aCGH (median OS: 71 *vs* 43 months) [37].

Finally, the somatic mutation analysis of *TP53, ASXL1, EZH2*, *TET2, RUNX1, DNMT3A and SF3B1* genes allowed us identifying different mutations in 54% of patients; in 22 patients (36% of total) the molecular one was the only identified marker of clonality. In patients with MDS, mutations in *TP53, EZH2, ETV6, RUNX1*, and *ASXL1* were found to be recurrent and to predict poor overall survival, independently of other established risk factors [38]. In our cohort, the more frequent somatic mutations included *TP53 and SF3B1.*

The *TP53*-mutated patients were principally in the low/intermediate-1 classes; one patient with ring sideroblastic anemia at low IPSS risk, with normal karyotype, FISH and aCG, presented SF3B1 and *TP53* mutations. Moreover, it is interesting to note that in our series we observed 3 patients *TP53*-mutated with a concomitant neoplasia. *TP53* mutations have been reported also in many hematological and solid tumors, even with adverse outcome [39-41]. In line with this results, in our cohort *TP53* mutations were detected in 3 cases of MDS arising after chemotherapies for previous lymphoproliferative disorders, and in the half of MDS patients with complex karyotype.

About the other molecular abnormalities found in our series, the number of the *TET2*-mutated patients is too small for deriving clinical conclusions; one patient well responded to azacitidine, as previously reported in literature [15], whereas the other one did not achieve satisfying results. Moreover, in our study 5 patients presented with *ASXL1* mutations; the small number of cases does not allow us achieving significant statistical results about its prognostic role. The negative prognostic role of *ASXL1* in acute and myeloproliferative disorders is now well recognized [42, 43], and the assessment of this gene mutations in the clinical practice could help physician to design from the diagnosis a patient-tailored treatment.

Other interesting findings coming from our study concern the assessment of *WT1* and *RPS14* gene expression. At the time of diagnosis, 70% of patients showed *RPS14* values lower than those measured in healthy donors, and 28% carried high *WT1* levels. Interestingly, OS was shorter for cases showing *WT1* higher values (18 months *versus* 41; *p* = 0.001), in analogy to what occurred for 30-months PFS (56% *versus* 89%; *p* = 0.01). To note that the 38% of our patients with high expression of *WT1* presented higher percentage of blasts, while 44% presented with cytogenetic alteration included in the intermediate and poor risk, as previously reported [24]. The negative impact of the *WT1* expression levels on survival in perfectly in accordance with data published by Nagasaki et al. who reported a negative impact of WT1 expression on OS in a series of old MDS patients (HR = 6.4) [44].

Lower *RPS14* expression in MDS patients without 5q deletion was reported to be associated with increased apoptosis of nucleated erythrocytes; consequently, an up-regulation during treatment could be a positively predictor marker of response to lenalidomide [45]. In our series, low *RPS14* levels were shown in more than 2/3 of patients. These data confirmed those from the literature, where reduced expression of *RPS14* was described in 71% of patients with intermediate IPSS risk in absence of chromosome 5 deletions [26]. Differently from these authors, who reported that PFS was positively conditioned by low *RPS14* levels, in our cohort the *RPS14* expression did not condition OS nor PFS even if a trend to longer PFS was observed in cases with low *RPS14* expression (30 months-PFS: 100% *versus* 78%; *p* = 0.13) receiving azacitidine.

In conclusion, our study supports the idea that an integrated diagnostics (cytogenetic and molecular) workup could be useful for a better risk stratification of MDS patients. The number of cases enrolled in our study is surely small, but it is the mirror of the real-life lived in the majority of hematological centres, where the diagnostic assays have to be optimized also for the small number of cases/month. Indeed, even in our center we employ an advanced NGS platform for detecting mutations in myeloid disorders; nevertheless, the optimization of times and costs implies at least 15-20 cases/run. Sometimes physicians need rapid results for choosing *ab initio* the more correct treatment; we suggest that in these cases simple real-time PCR assays as those presented in this study could answer this clinical need, with acceptable costs and time. The addition of FISH can ameliorate the cytogenetic definition of the risk score, with sustainable costs. Finally, the aCGH could even better define the risk of progression into acute leukemia, but this technique could be probably applied to cases with suboptimal response or failure for re-defining or modifying the therapeutic project instead of to all patients at diagnosis.

## PATIENTS AND METHODS

### Patients

A total of 61 patients (18 women and 43 men), with a median age of 74 years (range 30-92 years), were enrolled at time of diagnosis during a routinary control at the Hematology Unit of Pisa, from June 2012 to March 2015. All patients gave written informed consent for the use of their data for scientific evaluations.

According to the WHO classification, 33% were affected by multilineage refractory cytopenias (RCMD), 12% by refractory anemia (RA), 15% by refractory anemia with ring sideroblasts (RARS), 21% by refractory anemia with excess of blasts (RAEB 1-2), 3% by 5q- syndrome, and 12% by chronic myelo-monocytic leukemia (CMML).

### Conventional cytogenetics

Chromosomes were QFQ banded and at least 20 metaphases were analysed using standard procedures and karyotype was described, according to ISCN [28]. Structural chromosomal aberrations were considered if found in at least two metaphases. On the contrary, three complete metaphases presenting the same loss were required to diagnose a monosomy. Karyotype complexity (CK) was considered in accordance with Schanz et al (Schanz et al., 2012) including non-CK ( < three CAs), sCK (three CAs), and very CK ( > three CAs).

### FISH analysis

FISH analysis was performed according to standard protocols. FISH color probe sets included: 5p15.2 (normal range; 0-4%)/EGR1 (5q31) (Abbott Molecular, Abbott Park, IL), CEP7 (normal range; 0-5%)/7q31 (0-7%), CEP8 (normal range; 0-7%), 5q32 (PDGFRb-Breakapart. Cytocell Celbio) (normal range;0-5%) and 4q12 (FIP1L1/CHIC2/PDGFRa-deletion/fusion. Cytocell Celbio) (normal range; 0-5%). Thresholds for interpretation as a positive result were established for each probe at 3 standard deviations above the mean of 20 normal bone marrow samples.

### aCGH analysis

After DNA extraction from 200 uL of whole bone marrow by the QIAamp DNA Blood Mini Kit (Qiagen, Hilden, Germany), oligonucleotide whole genome array CGH (Agilent Human Genome Microarray Array-CGH) analysis was performed according to manufacturer’s protocol (Agilent Technologies, Paolo Alto CA) and the search for Copy Number Variation Regions (CNVRs) was performed using the Database of Genomic Variants (http://projects.tcag.ca/variation/).

### Somatic mutation analysis

Mutation detection research was performed using *qBiomarker Somatic Mutation PCR Arrays* (Qiagen); each array is comprised of a custom panel of gene mutations, including *TP53, ASXL1, EZH2*, *TET2, RUNX1, DNMT3A and SF3B1* genes, selected from comprehensive somatic mutation databases (COSMIC). The assay was established in a RT-PCR based on Amplification Refractory Mutation System (ARMS) technology. The panel included: a) seventeen different *TP53* gene mutations comprised on DNA binding domain and ranged from 163 to 285 aa; b) three different *ASXL1* gene mutations comprised on exon 12 and two frame-shift mutations in SCR1 binding domain; c) three *EZH2* gene mutations located in the SET domain, d) two *TET2* gene mutations comprised on exon 3, e) seven RUNX1 gene mutations identified in exons 3, 4 and 6, f) two DNMT3A gene mutations into methyltransferase domain and g) three different SF3B1 gene mutations clustered in the HEAT domain repeats (HD).

### WT1 and RPS14 gene expression analysis

After RNA extraction from bone marrow blood samples using RNeasy Mini Kit (QIAGEN, Valencia, CA, USA) and the cDNA synthesis performed using about 1 ug of RNA according to manufacturer’s protocol (Invitrogen, Thermo Fisher Scientific, Massachusetts, USA), different RT-PCR quantification analysis were performed for *WT1* and *RPS14* genes.

The *WT1* gene expression analysis were assessed by the *“WT1 ProfileQuant*^®^ *kit (ELN)”* (Ipsogen, Marseille, France), whose components have been tested in the context of a collaborative study of the European Leukemia Net consortium (ELN) and selected as the *ELN WT1 assay*. On the contrary, the RT-PCR quantitative assays for *RPS14* gene was performed using separated amplification of target gene and of *18S* gene as endogenous control. In each reaction were assessed standard serial dilutions of control gene and RPS14 plasmids, using a commercial kit primers design (Eppendorf). Moreover, cDNA samples from five 5q- syndromes and 6 healthy donors (patients aged 60-70 years who underwent femur surgery) will be used as positive and negative controls respectively.

### Statistical analysis

Overall Survival (OS) was calculated from the time of diagnosis to the last visit or follow-up or death; patients were censored at the moment of death or just before the allogeneic transplantation (in two cases).

Time to event variables (OS and progression-free-survival or PFS) are presented as Kaplan-Meier plots of time to first event.

Response rates will be expressed as percentages with their 95% Exact Clopper Pearson Confidence Interval limits. T-test has been employed for comparing median and mean values for continuous numerical variables.

To determine significances in categorized variables, SPSS 17.0 (SPSS Inc, Chicago, IL, USA) was used to calculate Pearson Chi-square or Fisher’s exact test, when appropriate. All tests were 2-sided. Results were considered statistically significant for p≤0.05.

## References

[1] Cazzola M, Malcovati L (2005). Myelodysplastic syndromes—coping with ineffective hematopoiesis. N Engl J Med.

[2] Greenberg P, Cox C, LeBeau MM, Fenaux P, Morel P, Sanz G, Sanz M, Vallespi T, Hamblin T, Oscier D, Ohyashiki K, Toyama K, Aul C (1997). International scoring system for evaluating prognosis in myelodysplastic syndromes. Blood.

[3] Mufti GJ, Bennett JM, Goasguen J, Bain BJ, Baumann I, Brunning R, Cazzola M, Fenaux P, Germing U, Hellström-Lindberg E, Jinnai I, Manabe A, Matsuda A (2008). Diagnosis and classification of myelodysplastic syndrome: international Working Group on Morphology of myelodysplastic syndrome (IWGM-MDS) consensus proposals for the definition and enumeration of myeloblasts and ring sideroblasts. Haematologica.

[4] Arber DA, Orazi A, Hasserjian R, Thiele J, Borowitz MJ, Le Beau MM, Bloomfield CD, Cazzola M, Vardiman JW (2016). The 2016 revision to the World Health Organization classification of myeloid neoplasms and acute leukemia. Blood.

[5] Malcovati L, Nimer SD (2008). Myelodysplastic syndromes: diagnosis and staging. Cancer Control.

[6] Greenberg PL, Tuechler H, Schanz J, Sanz G, Garcia-Manero G, Solé F, Bennett JM, Bowen D, Fenaux P, Dreyfus F, Kantarjian H, Kuendgen A, Levis A (2012). Revised international prognostic scoring system for myelodysplastic syndromes. Blood.

[7] Nybakken GE, Bagg A (2014). The genetic basis and expanding role of molecular analysis in the diagnosis, prognosis, and therapeutic design for myelodysplastic syndromes. J Mol Diagn.

[8] Romeo M, Chauffaille ML, Silva MR, Bahia DM, Kerbauy J (2002). Comparison of cytogenetics with FISH in 40 myelodysplastic syndrome patients. Leuk Res.

[9] Bernasconi P, Cavigliano PM, Boni M, Calatroni S, Klersy C, Giardini I, Rocca B, Crosetto N, Caresana M, Lazzarino M, Bernasconi C (2003). Is FISH a relevant prognostic tool in myelodysplastic syndromes with a normal chromosome pattern on conventional cytogenetics? A study on 57 patients. Leukemia.

[10] Costa D, Valera S, Carrió A, Arias A, Muñoz C, Rozman M, Belkaid M, Coutinho R, Nomdedeu B, Campo E (2010). Do we need to do fluorescence in situ hybridization analysis in myelodysplastic syndromes as often as we do?. Leuk Res.

[11] Slovak ML, Smith DD, Bedell V, Hsu YH, O’Donnell M, Forman SJ, Gaal K, McDaniel L, Schultz R, Ballif BC, Shaffer LG (2010). Assessing karyotype precision by microarray-based comparative genomic hybridization in the myelodysplastic/myeloproliferative syndromes. Mol Cytogenet.

[12] Bajaj R, Xu F, Xiang B, Wilcox K, Diadamo AJ, Kumar R, Pietraszkiewicz A, Halene S, Li P (2011). Evidence-based genomic diagnosis characterized chromosomal and cryptic imbalances in 30 elderly patients with myelodysplastic syndrome and acute myeloid leukemia. Mol Cytogenet.

[13] Kolquist KA, Schultz RA, Furrow A, Brown TC, Han JY, Campbell LJ, Wall M, Slovak ML, Shaffer LG, Ballif BC (2011). Microarray-based comparative genomic hybridization of cancer targets reveals novel, recurrent genetic aberrations in the myelodysplastic syndromes. Cancer Genet.

[14] Otrock ZK, Tiu RV, Maciejewski JP, Sekeres MA (2013). The need for additional genetic markers for myelodysplastic syndrome stratification: what does the future hold for prognostication?. Expert Rev Hematol.

[15] Kosmider O, Gelsi-Boyer V, Cheok M, Grabar S, Della-Valle V, Picard F, Viguié F, Quesnel B, Beyne-Rauzy O, Solary E, Vey N, Hunault-Berger M, Fenaux P, and Groupe Francophone des Myélodysplasies (2009). TET2 mutation is an independent favorable prognostic factor in myelodysplastic syndromes (MDSs). Blood.

[16] Gelsi-Boyer V, Trouplin V, Roquain J, Adélaïde J, Carbuccia N, Esterni B, Finetti P, Murati A, Arnoulet C, Zerazhi H, Fezoui H, Tadrist Z, Nezri M (2010). ASXL1 mutation is associated with poor prognosis and acute transformation in chronic myelomonocytic leukaemia. Br J Haematol.

[17] Ernst T, Chase AJ, Score J, Hidalgo-Curtis CE, Bryant C, Jones AV, Waghorn K, Zoi K, Ross FM, Reiter A, Hochhaus A, Drexler HG, Duncombe A (2010). Inactivating mutations of the histone methyltransferase gene EZH2 in myeloid disorders. Nat Genet.

[18] Paschka P, Schlenk RF, Gaidzik VI, Habdank M, Krönke J, Bullinger L, Späth D, Kayser S, Zucknick M, Götze K, Horst HA, Germing U, Döhner H (2010). IDH1 and IDH2 mutations are frequent genetic alterations in acute myeloid leukemia and confer adverse prognosis in cytogenetically normal acute myeloid leukemia with NPM1 mutation without FLT3 internal tandem duplication. J Clin Oncol.

[19] Kosmider O, Gelsi-Boyer V, Slama L, Dreyfus F, Beyne-Rauzy O, Quesnel B, Hunault-Berger M, Slama B, Vey N, Lacombe C, Solary E, Birnbaum D, Bernard OA (2010). Mutations of IDH1 and IDH2 genes in early and accelerated phases of myelodysplastic syndromes and MDS/myeloproliferative neoplasms. Leukemia.

[20] Kita-Sasai Y, Horiike S, Misawa S, Kaneko H, Kobayashi M, Nakao M, Nakagawa H, Fujii H, Taniwaki M (2001). International prognostic scoring system and TP53 mutations are independent prognostic indicators for patients with myelodysplastic syndrome. Br J Haematol.

[21] Patel JP, Gönen M, Figueroa ME, Fernandez H, Sun Z, Racevskis J, Van Vlierberghe P, Dolgalev I, Thomas S, Aminova O, Huberman K, Cheng J, Viale A (2012). Prognostic relevance of integrated genetic profiling in acute myeloid leukemia. N Engl J Med.

[22] Call KM, Glaser T, Ito CY, Buckler AJ, Pelletier J, Haber DA, Rose EA, Kral A, Yeger H, Lewis WH, Jones C, Housman DE (1990). Isolation and characterization of a zinc finger polypeptide gene at the human chromosome 11 Wilms’ tumor locus. Cell.

[23] Tamaki H, Ogawa H, Ohyashiki K, Ohyashiki JH, Iwama H, Inoue K, Soma T, Oka Y, Tatekawa T, Oji Y, Tsuboi A, Kim EH, Kawakami M (1999). The Wilms’ tumor gene WT1 is a good marker for diagnosis of disease progression of myelodysplastic syndromes. Leukemia.

[24] Cilloni D, Gottardi E, Messa F, Fava M, Scaravaglio P, Bertini M, Girotto M, Marinone C, Ferrero D, Gallamini A, Levis A, Saglio G (2003). Piedmont Study Group on Myleodysplastic Syndromes. Significant correlation between the degree of WT1 expression and the International Prognostic Scoring System Score in patients with myelodysplastic syndromes. J Clin Oncol.

[25] Raza A, Cruz R, Latif T, Mukherjee S, Galili N (2010). The biology of myelodysplastic syndromes: unity despite heterogeneity. Hematol Rep.

[26] Czibere A, Bruns I, Junge B, Singh R, Kobbe G, Haas R, Germing U (2009). Low RPS14 expression is common in myelodysplastic syndromes without 5q- aberration and defines a subgroup of patients with prolonged survival. Haematologica.

[27] Wall M, Rayeroux KC, MacKinnon RN, Zordan A, Campbell LJ (2012). ETV6 deletion is a common additional abnormality in patients with myelodysplastic syndromes or acute myeloid leukemia and monosomy 7. Haematologica.

[28] Shaffer LG, Tommerup N

[29] Galimberti S, Ferreri MI, Simi P, Azzarà A, Baratè C, Fazzi R, Cecconi N, Cervetti G, Guerrini F, Petrini M (2009). Platelet-derived growth factor beta receptor (PDGFRB) gene is rearranged in a significant percentage of myelodysplastic syndromes with normal karyotype. Br J Haematol.

[30] Lai YY, Huang XJ, Li J, Zou P, Xu ZF, Sun H, Shao ZH, Zhou DB, Chen FP, Liu ZG, Zhu HL, Wu DP, Wang C (2015). Standardized fluorescence in situ hybridization testing based on an appropriate panel of probes more effectively identifies common cytogenetic abnormalities in myelodysplastic syndromes than conventional cytogenetic analysis: a multicenter prospective study of 2302 patients in China. Leuk Res.

[31] Wong AK, Fang B, Zhang L, Guo X, Lee S, Schreck R (2008). Loss of the Y chromosome: an age-related or clonal phenomenon in acute myelogenous leukemia/myelodysplastic syndrome?. Arch Pathol Lab Med.

[32] Persani L, Bonomi M, Lleo A, Pasini S, Civardi F, Bianchi I, Campi I, Finelli P, Miozzo M, Castronovo C, Sirchia S, Gershwin ME, Invernizzi P (2012). Increased loss of the Y chromosome in peripheral blood cells in male patients with autoimmune thyroiditis. J Autoimmun.

[33] Dumanski JP, Lambert JC, Rasi C, Giedraitis V, Davies H, Grenier-Boley B, Lindgren CM, Campion D, Dufouil C, Pasquier F, Amouyel P, Lannfelt L, Ingelsson M (2016). Mosaic Loss of Chromosome Y in Blood Is Associated with Alzheimer Disease. Am J Hum Genet.

[34] Noveski P, Madjunkova S, Sukarova Stefanovska E, Matevska Geshkovska N, Kuzmanovska M, Dimovski A, Plaseska-Karanfilska D (2016). Loss of Y Chromosome in Peripheral Blood of Colorectal and Prostate Cancer Patients. PLoS One.

[35] De Braekeleer E, Douet-Guilbert N, Morel F, Le Bris MJ, Basinko A, De Braekeleer M (2012). ETV6 fusion genes in hematological malignancies: a review. Leuk Res.

[36] Padron E, Yoder S, Kunigal S, Mesa T, Teer JK, Al Ali N, Sekeres MA, Painter JS, Zhang L, Lancet J, Maciejewski JP, Epling-Burnette PK, Sotomayor E (2014). ETV6 and signaling gene mutations are associated with secondary transformation of myelodysplastic syndromes to chronic myelomonocytic leukemia. Blood.

[37] Volkert S, Haferlach T, Holzwarth J, Zenger M, Kern W, Staller M, Nagata Y, Yoshida K, Ogawa S, Schnittger S, Haferlach C (2016). Array CGH identifies copy number changes in 11% of 520 MDS patients with normal karyotype and uncovers prognostically relevant deletions. Leukemia.

[38] Haase D, Germing U, Schanz J, Pfeilstöcker M, Nösslinger T, Hildebrandt B, Kundgen A, Lübbert M, Kunzmann R, Giagounidis AA, Aul C, Trümper L, Krieger O (2007). New insights into the prognostic impact of the karyotype in MDS and correlation with subtypes: evidence from a core dataset of 2124 patients. Blood.

[39] Tausch E, Mertens D, Stilgenbauer S (2016). Genomic Features: Impact on Pathogenesis and Treatment of Chronic Lymphocytic Leukemia. Oncol Res Treat.

[40] Teoh PJ, Chng WJ (2014). p53 abnormalities and potential therapeutic targeting in multiple myeloma. Biomed Res Int.

[41] Gu J, Zhou Y, Huang L, Ou W, Wu J, Li S, Xu J, Feng J, Liu B (2016). TP53 mutation is associated with a poor clinical outcome for non-small cell lung cancer: evidence from a meta-analysis. Mol Clin Oncol.

[42] Tefferi A (2010). Novel mutations and their functional and clinical relevance in myeloproliferative neoplasms: JAK2, MPL, TET2, ASXL1, CBL, IDH and IKZF1. Leukemia.

[43] Bejar R, Stevenson K, Abdel-Wahab O, Galili N, Nilsson B, Garcia-Manero G, Kantarjian H, Raza A, Levine RL, Neuberg D, Ebert BL (2011). Clinical effect of point mutations in myelodysplastic syndromes. N Engl J Med.

[44] Nagasaki J, Aoyama Y, Hino M, Ido K, Ichihara H, Manabe M, Ohta T, Mugitani A (2017). Wilms Tumor 1 (WT1) mRNA Expression Level at Diagnosis Is a Significant Prognostic Marker in Elderly Patients with Myelodysplastic Syndrome. Acta Haematol.

[45] Oliva EN, Cuzzola M, Nobile F, Ronco F, D’Errigo MG, Laganà C, Morabito F, Galimberti S, Cortelezzi A, Aloe Spiriti MA, Specchia G, Poloni A, Breccia M (2010). Changes in RPS14 expression levels during lenalidomide treatment in Low- and Intermediate-1-risk myelodysplastic syndromes with chromosome 5q deletion. Eur J Haematol.

